# Laboratory Diagnostics of Botulism Cases in Livestock in Poland in 2022–2024

**DOI:** 10.3390/pathogens15030302

**Published:** 2026-03-10

**Authors:** Aleksandra Jarosz, Magdalena Sapała, Tomasz Grenda

**Affiliations:** Department of Food and Feed Microbiology, National Veterinary Research Institute, Partyzantów Ave. 57, 24-100 Puławy, Poland; magdalena.sapala@piwet.pulawy.pl

**Keywords:** animal botulism, botulinum neurotoxins, laboratory diagnostics, livestock

## Abstract

Botulism is a neuroparalytic disease caused by exposure to botulinum neurotoxins produced by anaerobic spore-forming bacteria of the genus *Clostridium*. This disease occurs in both humans and wild and domestic animals, and is currently becoming an increasingly serious problem worldwide due to high animal mortality and economic losses. The clinical signs observed during the progression of botulism are nonspecific and difficult to unequivocally associate with this disease entity. The aim of this study is to present laboratory diagnostics of suspected botulism cases reported in Poland in 2022–2024, as well as to present the challenges encountered during laboratory investigations. The material for the study consisted of samples of liver, serum, digestive tract, feed, feces, straw, and water from drinking lines, sent to the National Veterinary Research Institute (NVRI) in relation to thirteen suspected cases of botulism, predominantly reported in poultry, but also in mink and cattle farms. The samples were analyzed using a mouse bioassay and conventional culture methods, as well as real-time PCR methods aimed at detecting the *ntnh* and *bont* genes, which determine the production of botulinum neurotoxins. Of the thirteen suspected cases analyzed, ten were confirmed by the detection of botulinum toxin (BoNTs) and/or the presence of the *ntnh* and *bont* genes in the tested material. Based on the results obtained, it was concluded that botulinum toxin type C was the etiological factor of botulism poisoning in most of the analyzed cases. In one case reported in cattle, poisoning occurred as a result of the mosaic variant of BoNT D/C. Due to the nonspecific signs of botulism and the time required for them to appear, laboratory diagnostics play a key role in detecting the disease. However, this process is complicated due to the high heterogeneity observed among *Clostridium* spp. strains, as well as difficulties encountered during the isolation of the microorganism and the possibility of loss of toxin-producing capacity at later stages of analysis.

## 1. Introduction

Botulism is a neuroparalytic disease observed in humans, as well as wild and farm animals, caused by inhibition of acetylcholine release at neuromuscular junctions following exposure to botulinum neurotoxins (BoNTs), which are among the most potent naturally occurring toxins in the environment [1,2,3]. The ability to produce these toxins is most often attributed to *Clostridium botulinum*, which represents anaerobic, Gram-positive spore-forming bacteria. Although most species belonging to the genus *Clostridium* are saprophytic bacteria, the literature data indicate that botulinum neurotoxin production may also occur in other *Clostridium* spp., such as *C. baratii*, *C. butyricum*, and *C. sporogenes* [4,5,6,7]. These microorganisms are widespread in the environment, especially in soil, water sediments, and organic matter, where they can survive in spore form for decades, constituting a permanent microbiological source [8,9].

Currently, seven types of toxic BoNTs are recognized, designated by the letters A–G, as well as approximately 40 subtypes of these toxins [10,11,12]. Taking into account physiological properties and differences in the sequences of the conservative 16S rRNA gene, *C. botulinum* strains have been classified into four metabolic groups: group I includes toxin type A and proteolytic strains of types B and F, while group II includes non-proteolytic strains of types E, B, and F. The third group includes *C. botulinum* strains of types C and D and their mosaic variants C/D and D/C, while the fourth group includes strains of type G. It is believed that the etiological factors causing botulism in humans are *C. botulinum* strains capable of producing toxins A, B, E, and F, while in animals, the disease is most often caused by toxotypes C, D, and mosaic variants [13,14,15].

In recent years, botulism in livestock has gained importance as a problem of growing global importance. This disease is particularly relevant in intensive production systems, where its occurrence is associated with high animal mortality and considerable economic losses, especially on poultry farms, in fur farming, and in ruminants [16,17]. Botulism in animals can take the form of both classic intoxication, associated with the ingestion of feed or environmental material containing the toxin, and toxicoinfection resulting from the multiplication of bacteria in the gastrointestinal tract and the production of toxins in situ [10,18]. Both forms lead to a similar clinical picture including locomotor disorders, weakened reflexes, difficulty in swallowing, and progressive respiratory failure. In ungulates and fur animals, difficulty in maintaining balance is often observed, while in birds, characteristic drooping of the wing occurs, and in advanced stages, complete inability to move [19,20,21,22]. The time required for signs to appear is variable, depending on the dose of botulinum toxin. In classic intoxication, clinical signs may appear after several hours or after 1–3 days, while in toxicoinfections, the development of the disease is sometimes slower because the time needed to produce the toxin in situ is longer [10,11,17]. Differential diagnosis is often complicated because early clinical signs are often nonspecific and can be confused with other neurological, metabolic, or toxicological conditions, and the dynamics of the disease can lead to sudden deaths of entire groups of animals, which poses a major diagnostic challenge [16,23,24].

Due to the nonspecific character of the signs of pathogenesis, and a wide spectrum of potential sources of exposure, the diagnosis of botulism in livestock is extremely challenging. However, it also provides the basis for confirming the etiology of the disease through the detection and identification of botulinum toxin in the tested biological material. Accurate and rapid laboratory diagnostics are essential in suspected botulism outbreaks because the disease is caused by preformed botulinum neurotoxins rather than the mere presence of the bacteria. Detection of *Clostridium botulinum* or genes indicates the potential for toxin production, whereas direct detection of botulinum toxin confirms exposure of animals to biologically active toxin. These two diagnostic approaches therefore provide complementary information for clinical and epidemiological interpretation of outbreaks. Rapid diagnosis is particularly important because the effectiveness of antitoxin therapy depends on early neutralization of circulating toxin before irreversible neuromuscular damage occurs. In addition, laboratory confirmation of botulism plays an important epidemiological role, as the identification of toxin types and potential environmental sources of contamination allows the implementation of appropriate control measures in livestock production systems [23,25]. However, the interpretation of diagnostic results should be performed with caution, as both false-positive and false-negative findings may occur depending on the analyzed matrix, the stage of disease progression, and the diagnostic method applied.

The aim of this study is to present laboratory diagnostics of suspected cases of botulism in livestock recorded in Poland between 2022 and 2024, and to discuss the key challenges associated with the laboratory diagnosis of botulism in animals.

## 2. Materials and Methods

### 2.1. Material Intended for Research

The material for the study consisted of samples of liver, serum, digestive tract, feed, feces, straw, and water from drinking lines sent to the National Veterinary Research Institute (NVRI) in Puławy between 2022 and 2024 in association with all suspected cases of botulism in livestock that were officially reported and submitted for laboratory confirmation. Therefore, the number of cases analyzed reflects the complete national dataset available for this time period and was not subject to selection or exclusion criteria. The samples for examination were submitted together with standardized questionnaires containing background information used for further analysis. The study was based on independent diagnostic reports, and no longitudinal monitoring or quantitative assessment of toxins was performed. Observation of affected animals was carried out by veterinary authorities and was beyond the scope of the laboratory’s diagnostic activities. The most frequently observed signs included: decreased muscle tone, breathing difficulties, drooping eyelids, drooping neck, drooling, and flaccid paralysis. The majority of suspected cases of botulism involved poultry, particularly chickens and turkeys, with occasional reports of suspected botulism in cattle and mink. In total, 42 samples were included in the analysis. Depending on the sample type and its suitability for a given diagnostic approach, different laboratory methods were applied. Molecular analyses were used as a primary screening tool, whereas the mouse bioassay (MBA) was performed exclusively on serum samples. Data on individual cases, including animal species, place and time of occurrence, type of samples tested, and average air temperature during that period are presented in Table 1. Additionally, data on the number of affected animals and observed clinical signs in individual cases are presented in Table 2.

### 2.2. Mouse Bioassay (MBA)

The mouse bioassay was performed exclusively on selected serum samples and was not applied to environmental or feed matrices. The MBA test was conducted after obtaining approval from the Local Ethics Committee in Lublin (approval no. 53/2021), in accordance with the protocol of the US Food and Drug Administration [26] and the Polish standard PN-R-64791:1994 [27]. Suspensions were prepared using phosphate buffer in a 1:1 ratio, and then injected intraperitoneally into mice in a volume of 0.2 mL. In order to determine the presence of botulinum toxin in the tested material, characteristic signs of botulism were observed, such as constipation, progressive paralysis of the limbs, difficulty breathing, and wasp waist. A positive result was confirmed by a seroneutralization test using botulinum antitoxins. The aforementioned test was performed by intraperitoneal injection of type-specific botulinum antitoxins obtained from the National Institute for Biological Standards and Control (NIBSC, South Mimms, UK), according to the manufacturer’s instructions. After 20 min, the animals were reinjected intraperitoneally with the tested material. Neutralization of toxin activity confirmed the presence of the corresponding botulinum toxin type.

### 2.3. Culture Methods

Depending on the quantity, the examined samples were inoculated into bottles containing 10 mL to 90 mL of TPGY medium regenerated to remove oxygen in a water bath at 100 °C for 15 min (50 g/L casein enzymic hydrolysate, 5 g/L peptic digest of animal tissue, 20 g/L yeast extract, 4 g/L dextrose, and 1 g/L sodium thioglycolate, with a final pH of 7.0 ± 0.2 at 25 °C) in a ratio of 1:9 in two replicates, one of which was pasteurized at 70 °C for 15 min to select for spore-forming bacteria, including *Clostridium* spp., as bacterial spores are resistant to heat treatment while vegetative cells are eliminated. The inocula prepared in this way were incubated at 37 °C for 48 h in an anaerobic atmosphere obtained by using appropriate anaerobic chambers, atmosphere generating sachets (AnaeroGen, Thermo Scientific, Waltham, MA, USA) and test strips for detecting anaerobic atmosphere (Merck Millipore, Darmstadt, Germany).

After the incubation period, approximately 10 µL of each culture was spread on Willis-Hobbs (10 g/L peptic digest of animal tissue, 10 g/L meat extract, 5 g/L sodium chloride, 12 g/L lactose, 0.032 g/L neutral red, 10 g/L skim milk powder, 2 g egg yolk powder and 10 g/L agar, with a final pH of 7.0 ± 0.2 at 25 °C) and FAA (23 g/L peptone, 5 g/L sodium chloride, 1 g/L soluble starch, 0.4 g/L sodium bicarbonate, 1 g/L glucose, 1 g/L sodium pyruvate, 0.5 g/L L-cysteine HCl H_2_O, 0.25 g/L sodium pyrophosphate, 1 g/L L-arginine, 0.5 g/L sodium succinate, 0.01 g/L hemin, 0.001 g/L vitamin K, 2 g/L egg yolk powder and 12 g/L agar, with a final pH of 7.2 ± 0.2 at 25 °C) agar media. The prepared plates were incubated at 37 °C for 48 h using the above-mentioned anaerobic conditions. The colonies were evaluated for shape, size, surface and observed lipolytic, proteolytic or lecitinolytic features. All methods used in this study have been previously described, optimized, and approved in accordance with PN-EN-ISO/IEC 17025:2017 [28]. Colonies suspected of belonging to *Clostridium botulinum* were subcultured and subjected to PCR detection of the *ntnh* and *bont* genes to confirm their toxigenic potential.

### 2.4. DNA Isolation

DNA was isolated from 1 mL of liquid cultures and selected colonies obtained on agar media. Genetic material was extracted using the Genomic Mini AX Bacteria kit (A&A Biotechnology, Gdynia, Poland) in accordance with the manufacturer’s instructions. The amount of DNA was estimated using a Nicolet Evolution 300 spectrophotometer (Thermo Fisher Scientific, Waltham, MA, USA). The extracted DNA was frozen at −20 °C for further analysis or directly subjected to PCR reactions.

### 2.5. Detection of the ntnh Gene by Real-Time PCR

The DNA isolated in the previous stage was used to detect genes responsible for the production of botulinum neurotoxins. The *ntnh* gene encodes a non-hemagglutinin component of botulinum protoxin and is a characteristic element of the botulinum gene cluster of all *Clostridium* spp. strains capable of producing BoNTs. For this purpose, a set of seven primers and a TaqMan probe were used in accordance with the method described by Raphael et al. [29] The reaction mixture consisted of 5 µL of DNA, 4 µL of LightCycler TaqMan Master (Roche, Basel, Switzerland), 0.7 µM of each primer, and 0.24 µM of NTNH410 TaqMan probe. The LightCycler 2.0 thermocycler (Roche, Basel, Switzerland) was used for the analysis, with the following temperature profile: initial denaturation at 95 °C for 10 min and 40 cycles of denaturation at 95 °C for 15 s, annealing at 42 °C for 15 s and elongation at 55 °C for 1 min. As the control samples, DNA isolated from the following reference strains was used: *C. botulinum* NCTC 887 (type A), *C. botulinum* NCTC 3815 (type B), *C. botulinum* NCTC 8266 (type E) and *C. botulinum* NCTC 10281 (type F).

### 2.6. Detection of bont Genes by Real-Time PCR

After obtaining positive results, real-time PCR reactions were performed to detect *bont* genes for *Clostridium botulinum* group III, encoding the active component of botulinum toxins responsible for botulism in animals, according to the methodology described by Anniballi et al. [30]. The reaction mixtures consisted of 5 µL of DNA, 4 µL of LightCycler TaqMan Master (Roche, Basel, Switzerland), 0.7 µM of each primer, and 0.24 µM of each probe. The reactions were performed using a LightCycler 2.0 thermocycler (Roche, Basel, Switzerland) with the following temperature profile: initial denaturation at 95 °C for 15 min, followed by 40 cycles of denaturing at 94 °C for 30 s and annealing extension at 56 °C for 90 s. The control samples consisted of DNA obtained from strains of *C. botulinum* type C, *C. botulinum* type D, and *C. botulinum* types C/D and D/C included in the National Veterinary Research Institute’s own collection.

## 3. Results

### 3.1. Case Interpretation

**Figure 1:**
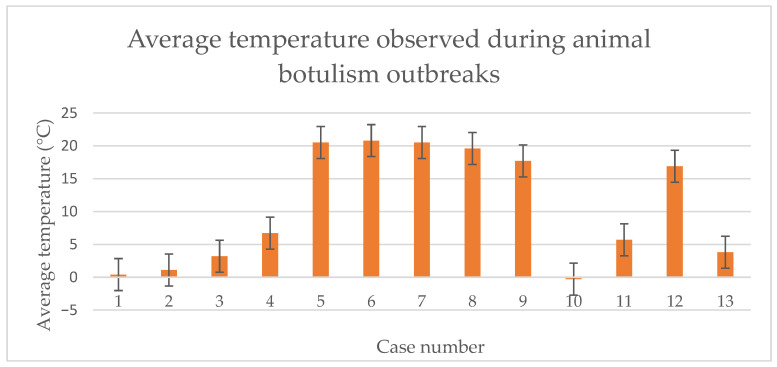
The average air temperatures recorded during the botulism cases.

Between 2022 and 2024, the National Veterinary Research Institute examined thirteen cases of suspected botulinum toxin poisoning in livestock. Eleven cases were reported on poultry farms, while the remaining two concerned mink and cattle farms. These cases occurred in five provinces of Poland (Table 1), which constitute the largest livestock production in the country. During the summer season from June to September, six suspected cases of botulism were reported, with average air temperatures ranging from 16.9–20.8 °C, two cases were reported in spring, when the air temperature was 3.2 °C and 6.7 °C, and one case occurred in autumn with an average temperature of 3.8 °C. In addition, four suspected cases of botulism were also reported in winter, when temperatures ranged from −0.3 °C to 5.7 °C. The average air temperatures recorded during the occurrence of the cases of botulism are presented in Figure 1, according to data provided by the Institute of Meteorology and Water Management [31].

**Figure 2:**
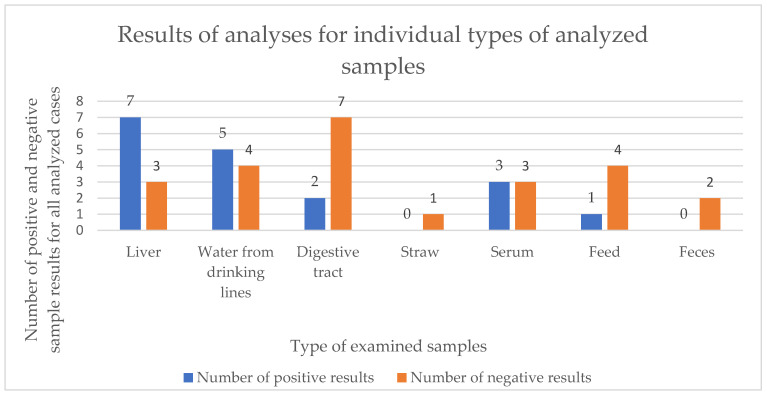
Test results depending on the type of matrix analyzed.

Samples from suspected cases were subjected to laboratory diagnosis using a mouse bioassay, as well as PCR methods aimed at detecting botulinum toxin genes. The results of these tests for individual samples and cases are presented in Table 3. Of the thirteen suspected cases of botulism analyzed, ten were confirmed by laboratory tests, and botulinum toxin type C was identified as the etiological factor in most cases. Only one case of botulism in cattle was caused by the D/C mosaic variant. The highest number of positive results was obtained for liver and drinking water samples, whereas negative results were obtained predominantly in digestive tracts, feeds, and manure samples. The test results obtained, depending on the matrix analyzed, are presented in Figure 2.

### 3.2. Culture Identification

From the liquid cultures of the tested samples, eleven strains suspected of belonging to the *Clostridium botulinum* species were isolated on Willis-Hobbs and FAA agar media, in which the characteristic “pearl layer” was observed, indicating the lipolytic properties of the isolates.

### 3.3. Mouse Bioassay Results

The mouse bioassay was performed on serum samples associated with six cases of botulism in poultry. Signs characteristic of this disease were observed in three serum samples (50%), while the MBA test was negative for the remaining three (50%). The seroneutralization test performed on positive samples allowed the toxins present in the serum samples to be classified as type C, which could be neutralized with an antitoxin dose of 1 IU.

### 3.4. Real-Time PCR Analysis Results

Real-time PCR reactions showed the presence of the *ntnh* gene in 18/36 samples analyzed using this method (50%). In addition, all eleven isolated strains were subjected to PCR analysis, which confirmed the presence of the *ntnh* and *bont* genes, indicating their toxigenic potential at the genetic level. Depending on the biological material examined, the *ntnh* gene was found in the following samples: liver (7/10), drinking water (5/9), digestive tract (2/9), and feed (1/5). No *ntnh* or *bont* genes responsible for the production of botulinum neurotoxins were found in samples associated with botulism cases 3, 7, and 8. In addition, the results of real-time PCR targeting the detection of individual *bont* genes showed the presence of the *bont*/C gene in samples associated with cases of botulism in poultry and mink (9/13 of the cases analyzed) and the *bont*/DC gene in a suspected case in cattle (1 case).

## 4. Discussion

Botulism is considered one of the most serious diseases affecting livestock, caused by exposure to botulinum toxins produced during the germination of *Clostridium botulinum* spores. Although this disease is becoming increasingly common in animal husbandry around the world, it remains largely misunderstood and, in addition to threatening animal health, also poses a risk of enormous economic losses [32,33].

In this study, we demonstrate the laboratory diagnosis of 13 suspected cases of botulism reported in Poland between 2022 and 2024, mainly on poultry farms, but also on cattle and mink farms. It should be emphasized that the present study is diagnostic and descriptive in nature. Although the number of analyzed cases does not allow for epidemiological inference, it represents all suspected botulism cases officially investigated in livestock in Poland during the study period. Furthermore, the set of samples submitted for laboratory examination differed between individual cases because material collection was performed by field veterinarians during outbreak investigations and depended on the clinical situation on the farm. Of these, 10/13 (77%) cases were confirmed by detecting *C. botulinum* spores and identifying botulinum toxins in the tested material. Due to the non-specific nature of botulism signs, the difficulty in recognizing them and clearly linking them to this disease, and the varied time of their occurrence, laboratory diagnosis of suspected botulism plays a key role in recognizing this disease and taking appropriate action. However, this is a complex task due to the high heterogeneity observed among *Clostridium* spp. strains, but also due to the lack of appropriate culture media and the possibility of loss of toxin-producing capacity at various stages of analysis [21]. Currently, the gold standard in botulism diagnosis is still considered to be the MBA, which is characterized by high sensitivity and the ability to observe signs typical of this disease. However, its use is associated with well-recognized ethical concerns. In the present study, the application of the MBA was deliberately limited to selected serum samples, in which it is not possible to detect *C. botulinum* spores, as in the case of other methods used, but only the active toxin present in the tested material. This approach reflects current veterinary diagnostic practice, in which the use of animal testing is minimized and restricted to cases where alternative methods are insufficient. Performing this test requires proper preparation of the material by suspending it in a phosphate buffer to prevent rapid degradation of the botulinum toxin. An additional advantage is the possibility of using a seroneutralization test with botulinum antitoxins, which allows false positive results to be ruled out [19,34]. A comparison of the diagnostic results obtained for different sample matrices showed that samples from the liver and drinking water yielded the highest percentage of positive results, while gastrointestinal contents, feed, and feces more often yielded negative results. However, these observations should be interpreted cautiously, as the available sample matrices differed between cases and therefore do not represent a direct comparison of diagnostic sensitivity. Nevertheless, the results may provide practical indications regarding the diagnostic usefulness of certain matrices in routine investigations. The effectiveness of using the liver in laboratory diagnostics was also confirmed in studies conducted by Le Marechal et al. [34,35], which confirmed that this organ, when used for testing, revealed the highest number of botulism cases compared to other tested materials such as the spleen, cloacal swabs, and intestinal contents. In the case of water samples from drinking lines, a high number of positive results may indicate contamination, which may occur as a result of contact between animal feces or litter and drinking troughs, leading to further contamination of the breeding facility, whereby water may act as a carrier of spores, facilitating their detection by molecular biology methods. For this purpose, PCR techniques are commonly used, which are characterized by high sensitivity and specificity [17]. The combination of molecular methods and a mouse bioassay provided complementary diagnostic information. While real-time PCR enabled sensitive detection of toxin-related genes in a variety of matrices, the MBA confirmed the presence of biologically active toxin in selected serum samples. The study aimed to demonstrate their complementary roles in routine veterinary diagnostics.

Based on the results obtained from mouse bioassay and real-time PCR, it was found that in most of the analyzed cases of botulism, the causative agent recorded on poultry and mink farms was botulinum toxin type C, while botulism in cattle was caused by the mosaic variant BoNT/DC, which is the first case of this type in Poland. The predominance of type C in poultry and mink observed in this study is consistent with the known host association of this toxinotype. Type C is frequently linked to avian botulism and outbreaks in fur animals. While environmental conditions favorable for anaerobic bacterial growth may support the development of various toxinotypes, host susceptibility and species-specific epidemiological patterns likely contribute to the observed dominance of type C in these animal populations. Taking into account the data on animal mortality included in some questionnaires accompanying the samples sent to the National Veterinary Research Institute, the reported cases were of a both larger outbreaks, mainly on poultry and mink farms, where mortality reached up to 130,000 birds and 800 minks, as well as incidental cases, as evidenced by the case of botulism in cattle, where only two dead animals were reported.

Very often, the occurrence of botulism outbreaks in animals is influenced by prevailing weather conditions, particularly temperature. Warmer seasons may favor the growth of *C. botulinum* and the production of botulinum toxins in an anaerobic environment rich in organic matter, potentially leading to intoxication in animals [36,37,38]. Among the suspected cases of botulinum toxin poisoning analyzed, six outbreaks were reported in summer and early spring, when the average air temperature ranged from 16.9 °C to 20.8 °C, which may have created favorable environmental conditions for the growth of *C. botulinum* and the spread of spores in the animal environment. However, a significant proportion of cases were also reported in winter, when the average air temperature ranged from −0.3 °C to 6.7 °C. Although lower temperatures generally limit environmental toxin production, it cannot be excluded that different epidemiological mechanisms were involved in these cases. This interpretation remains hypothetical, as direct differentiation between intoxication and toxicoinfection was not performed in this study.

Botulism in cattle occurs sporadically and is often associated with the consumption of feed contaminated with botulinum toxin or *C. botulinum* spores, which is facilitated by the anaerobic conditions inside silos, as well as improper silage preparation, which depends on the pH of the acidification process, the presence of dry matter, and the fermentation process [39]. Occasionally, botulism in cattle may also be the result of cross-contamination occurring in animal husbandry, as described by Souillard et al. [40]. The authors presented a description of a mass outbreak of botulism caused by botulinum toxin type D/C, resulting from contamination of manure from broiler chickens, which highlights the need to maintain adequate biosecurity in animal husbandry.

There is currently a wealth of literature describing the problem of botulism on poultry farms around the world. Souillard et al. [41] described 17 cases of botulism on poultry farms in France between 2011 and 2013. These cases were linked to the presence of *C. botulinum* types C and D in all areas examined, including the poultry house, cloakroom, ventilation system, animal environment, and feed and water tanks, making it possible to identify critical points of contamination on the farms. Other cases of poultry botulism caused by botulinum toxin type C were also found in Central Africa and the Midwest [42,43,44]. The predominance of type C observed in the present study is consistent with the findings reported by Souillard et al., where type C and D were detected in multiple environmental compartments on poultry farms. Similarly, in our material, type C was the dominant toxinotype in poultry cases, suggesting comparable epidemiological patterns. However, in contrast to the detailed environmental mapping performed in the French study, our investigation was limited to diagnostic samples submitted for laboratory confirmation, which may explain differences in the scope of environmental assessment. On poultry farms, botulinum toxin is most often produced and accumulated in an anaerobic environment rich in organic matter. Certain animal behaviors, such as litter pecking and coprophagia, may further increase the risk of spreading *C. botulinum* spores and botulism to other individuals. Insects, which are themselves insensitive to botulinum toxins and can therefore be an ideal vector for their transmission, also play a special role in the etiology of botulism [13,33,45].

Taking the above into account, strengthening biosecurity measures is particularly important in intensive poultry and fur animal production systems in Poland, where high animal density and environmental accumulation of organic material may facilitate the persistence of spores. Effective cleaning and disinfection of premises and installations, control of feed storage conditions, proper management of bedding, separation of sick animals, and rapid removal of carcasses are essential measures that may significantly reduce the risk of botulism outbreaks in livestock farms [46,47].

## 5. Conclusions

Botulism poses a serious threat to livestock health, is reported worldwide, and laboratory diagnostics play a key role in its diagnosis. It can be based on the use of the gold standard, which is a mouse bioassay, culture methods, and molecular biology methods dedicated to the detection of *Clostridium botulinum* in the tested material or genes determining the production of botulinum toxins. Laboratory diagnosis of botulism is a complex task due to the high heterogeneity of *Clostridium* spp. strains and the difficulties encountered at various stages of the analysis, which necessitates standardization of laboratory procedures for tested material and strategies for managing botulism in animals. The presented studies describe the methods used in the diagnosis of thirteen suspected cases of botulism in livestock recorded in 2022–2024, a significant percentage of which (77%) were confirmed by laboratory tests, including the identification of the types of toxins responsible for the occurrence of botulism. These cases were reported in both warm and cold seasons, suggesting that botulism occurs both as food poisoning and toxicoinfection. The presented research results also emphasize the need to apply appropriate biosecurity rules in animal husbandry, which may contribute to reducing the risk of botulism cases.

## Figures and Tables

**Table 1:** Cases of suspected botulism in livestock in Poland between 2022 and 2024.

Case Number	Province	Animal Species/Number of Affected Animals	Type of Samples Examined	Month/Year	Average Temperature
1	Masovia	Turkey/N/S	Liver, water from drinking lines	January/2022	0.4 °C
2	Masovia	Broiler chicken/130,000	Digestive tract, straw, serum, liver, water from drinking lines	January/2022	1.1 °C
3	Masovia	Broiler chicken/N/S	Water from drinking lines	March/2022	3.2 °C
4	Kuyavian- Pomeranian	Broiler chicken/2000	Digestive tract, serum, water from drinking lines, liver	April/2022	6.7 °C
5	Masovia	Turkey/N/S	Liver, feed, water from drinking lines, feces, serum, digestive tract	August/2022	20.5 °C
6	Masovia	Turkey/N/S	Water from drinking lines, serum, liver	August/2022	20.8 °C
7	Warmia- Masuria	Turkey/N/S	Serum, liver, digestive tract	August/2022	20.5 °C
8	Łódź Province	Turkey/N/S	Water from drinking lines, serum	August/2023	19.6 °C
9	Lublin	Broiler chicken/N/S	Feed, water from drinking lines, digestive tract, liver	September/2023	17.7 °C
10	Lublin	Minks/800	Feed, liver, digestive tract	January/2024	−0.3 °C
11	Masovia	Broiler chicken/3000	Digestive tract	February/2024	5.7 °C
12	Lublin	Broiler chicken/N/S	Feed, water from drinking lines, digestive tract, liver	September/2024	16.9 °C
13	Masovia	Cattle/2	Liver, feces, digestive tract, TMR feed	November/2024	3.8 °C

**Table 2:** Number of affected animals and signs observed in individual cases.

Case Number	Clinical Signs Observed During the Outbreaks					
	Decreased Muscle Tone	Breathing Difficulties	Drooping Eyelids	Drooping Neck	Drooling	Flaccid Paralysis
1	+	−	−	−	−	+
2	+	+	+	−	−	−
3	−	−	−	−	−	+
4	+	+	−	+	−	+
5	−	−	−	−	−	+
6	−	−	−	−	−	+
7	−	−	−	−	−	+
8	−	−	−	−	−	+
9	+	+	−	+	−	+
10	+	+	+	−	+	+
11	−	−	−	−	−	+
12	+	−	−	+	−	−
13	N/S					

**Table 3:** Real-time PCR and MBA test results for individual samples analyzed.

Case Number	Type of Samples Examined	Isolation of *Clostridium botulinum*-like Strains	Results of Individual Analyses			Confirmed Botulinum Toxin Toxotype
			Mouse Bioassay	Detection of *ntnh* Gene	Detection of *bont* Genes	
1	Liver	+	N/A	+	+	Type C
	Water from drinking lines	−	N/A	+	+	
2	Digestive tract	−	N/A	−	−	Type C
	Straw	−	N/A	−	−	
	Serum	N/A	−	N/A	N/A	
	Liver	−	N/A	−	−	
	Water from drinking lines	+	N/A	+	+	
3	Water from drinking lines	−	N/A	−	−	−
4	Digestive tract	+	N/A	+	+	Type C
	Serum	N/A	+	N/A	N/A	
	Water from drinking lines	−	N/A	−	−	
	Liver	+	N/A	+	+	
5	Liver	+	N/A	+	+	Type C
	Feed	−	N/A	−	−	
	Water from drinking lines	−	N/A	+	+	
	Feces	−	N/A	−	−	
	Serum	N/A	+	N/A	N/A	
	Digestive tract	−	N/A	−	−	
6	Water from drinking lines	−	N/A	+	+	Type C
	Serum	N/A	+	N/A	N/A	
	Liver	+	N/A	+	+	
7	Serum	−	−	−	−	−
	Liver	−	N/A	−	−	
	Digestive tract	−	N/A	−	−	
8	Water from drinking lines	−	N/A	−	−	−
	Serum	−	−	−	−	
9	Feed	−	N/A	−	−	Type C
	Water from drinking lines	+	N/A	+	+	
	Digestive tract	−	N/A	−	−	
	Liver	+	N/A	+	+	
10	Feed	−	N/A	+	+	Type C
	Liver	−	N/A	−	−	
	Digestive tract	−	N/A	−	−	
11	Digestive tract	+	N/A	+	+	Type C
12	Feed	−	N/A	−	−	Type C
	Water from drinking lines	−	N/A	−	−	
	Digestive tract	−	N/A	−	−	
	Liver	+	N/A	+	+	
13	Liver	+	N/A	+	+	Type D/C
	Feces	−	N/A	−	−	
	Digestive tract	−	N/A	−	−	
	TMR feed	−	N/A	−	−	

## Data Availability

All relevant data are provided in the present manuscript.

## Abbreviations

The following abbreviations are used in this manuscript:

BoNTs	Botulinum neurotoxins
MBA	Mouse bioassay
N/A	Not applicable
N/S	Not specified
PCR	Polymerase Chain Reaction
